# Isolation and expression analysis of Salt Overly Sensitive gene family in grapevine (*Vitisvinifera*) in response to salt and PEG stress

**DOI:** 10.1371/journal.pone.0212666

**Published:** 2019-03-19

**Authors:** Yuanchun Ma, Li Wang, Jiaoyang Wang, Yan Zhong, Zong-Ming (Max) Cheng

**Affiliations:** 1 College of Horticulture, Nanjing Agricultural University, Nanjing, Jiangsu province, The People’s Republic of China; 2 Department of Plant Sciences, University of Tennessee, Knoxville, United States of America; Estacion Experimental del Zaidin, SPAIN

## Abstract

Salt stress is one of the major environmental constraints for the production and yield of grape (*Vitis vinifera*) worldwide. The SOS3 gene family is part of the Salt Overly Sensitive (SOS) signaling pathway, a well-defined signaling pathway known to play a role in plant response to salt stress. In this study, the grapevine SOS3 gene family was annotated and the role of the annotated genes in salinity stress response was characterized. Nine grapevine SOS3 genes was identified in the grapevine genome and was subsequently analyzed. The expression patterns of the nine *VviSOS3* genes, as determined by reverse transcription quantitative PCR (RT-qPCR), varied greatly in leaves, roots, and stems of in-vitro grown Pinot noir grapevine cultivar(PN40024) in response to salt (250mM NaCl) and polyethylene glycol 6000 (PEG, osmolality equal to the salt treatment) treatments over a 36h time period. All of the *VviSOS3* genes, except *VviSOS3*.*7*, were up-regulated in leaves in response to the salt and PEG treatments. The majority of *VviSOS3* genes, except *VviSOS3*.*8* were up-regulated in roots in response to the PEG stress, with an opposite expression pattern in the root and stem in response to salt stress. The salinity treatment decreased the soluble protein content. Based on the expression pattern and physiological data, *VviSOS3*.*7* and *VviSOS3*.*8* were identified as candidate genes for further functional characterizations regarding their role in the response of grapevine to salt stress.

## Introduction

The production of grapes is economically important worldwide. It is, however, subject to many abiotic stresses (e.g. heat, salinity and drought) during its production cycle[1]. These abiotic stresses can greatly limit crop yields and reduce quality [2,3]. High salinity is mainly due to the increase of Na+ and Cl-, causing hyperosmotic and infiltration conditions of soil solution, hindering the absorption of water and nutrients by plants [4,5]. Soils with a high level of salinity affect plant growth, resulting in hyperosmotic stress, ion imbalance, and oxidative damage to cells [6]. In order to deal with the harm caused by salt stress, selection and development of salt-resistant cultivars and rootstocks possessing higher ability to cope with longer salt stress in viticulture [7,8]. Different grape varieties have different tolerance to salinity.Nayer Mohammadkhani et al. screened 18 grape genotypes, selected 'H6', 'Gha-rashani', and belonged to salt-tolerant varieties, while 'Shirazi' and 'Ghezel Zuum' belonged to salt-sensitive varieties [9]. Plants also produce various osmolytes and other stress-response proteins that function to re-establish ionic and osmotic homeostasis and prevent salt-related injury to cellular structures, respectively [10–13]. Although there are many studies on the drought-grapevine relation there is limited information about the effect of salinity on the physiology and the gene regulaion for grapevine.

The Salt Overly Sensitive (SOS) signaling pathway is a classical signal pathway, when plant were exposed to high levels of NaCl [14,12] and functional in regulating osmotic homeostasis in plants in response to salt stress. The SOS pathway, which is involved in salt stress response, begins with SOS3, a myristoylat The SOS pathway is a ed protein with three EF hands for calcium binding. These EF hands physically interact with SOS2, a serine/threonine protein kinase (STK). There are three key components, SOS3 (a Ca^2+^ sensor of the CBL family, Calcineurin B-like) and SOS2 (a CIPK protein), which had been studied in grape [15], and SOS1 (a Na^+^/H^+^ antiporter), that have been well characterized for their roles in maintaining ion homeostasis during salt stress [16–18]. Single or combination of overexpression of SOS pathway genes can enhance salt tolerance in transgenic plants [16,19,20]. Additionally, the SOS pathway, in response to salinity stress, is also functionally conserved in plants [21,22]. The downstream target of the *SOS3-SOS2* kinase complex is *SOS1*, a plasma membrane Na^+^/H^+^ antiporter that exports Na^+^ from the cell. This pathway was first reported by characterizing genes whose expression was overly-sensitive to salt stress [23]. Recently, the SOS pathway has been reported in many species, such as in *Brassica juncea* [24], in Kochia scoparia [25] in wheat [26] and in Barley [27]. Ion homeostasis in plants is maintained by restricting the accumulation of sodium [28]. SOS signaling pathway has not been clarified in grapevine in response to salt stress. In SOS signal pathway, *SOS3* plays an essential role in salt tolerance in plants [23] and belongs to a unique family of genes that encode calcium-binding proteins with an EF-hand type calcium-binding motif [29,12].The most thorough studies on the mechanism of salinity tolerance in plants have been reported in *Arabidopsis* [30–32]. *SOS3* has been demonstrated to play a critical role in plant response to low levels of salt stress by mediating calcium-dependent microfilament (MF) reorganization [32]. Salt treatments have a greater impact on MF reorganization in *SOS3* mutant than in wild-type (WT) plants, and the damage occurs more rapidly and is more severe [31]. Over-expression of *SOS3* has also been shown to enhance plant salt tolerance [33].

Therefore, the main objective of the present study was to annotate the *SOS3* gene family in grapevine and to characterize their expression in response to salt stress. Changes in relative water content, levels of soluble proteins and proline, and the K^+^/Na^+^ ratio were also characterized.

## Materials and methods

### Identification and bioinformatics analysis of putative *VviSOS3* proteins in grapevine

The sequences of *Arabidopsis* SOS3 proteins were retrieved from the *Arabidopsis* information resource 10 genome release (http://www.arabidopsis.org) and used as queries to conduct a blast search against the *V*. *vinifera* proteome (12X genome coverage, release V1) at the grape genome website (http://genomes.cribi.unipd.it/grape/). All hits obtained from the blast search were further analyzed using Pfam (http://pfam.janelia.org) to determine whether or not they possessed an EF-hand pair domain (PF134999). The sequences with conserved EF-hand pair domains were considered as grapevine *SOS3* (*VviSOS3*) genes. Because all CBL proteins are characterized by four common helix-loop-helix structural motifs (the EF-hand) that could act as a calcium-binding site, and the spacing of each EF-hand motif as well as the numbers is absolutely conserved [34]. The final, non-redundant SOS3 protein sequences were used in further analyses. Sequence length, molecular weight, isoelectric point of the deduce polypeptides, and the N-terminal myristoylation site numbers were calculated for each peptide using tools on the ExPasy website (http://web.expasy.org/protparam/).

The sequences of the putative grapevine *VviSOS3* genes (*VviSOS3s*) were aligned based upon their translated protein sequence using Clustal X 2.1 [35] with default settings. The phylogenetic trees were constructed using MEGA 5.0 [36] using the neighbour-joining (NJ) method, and bootstrap values were calculated for 1000 replicates. All nine of the identified *VviSOS3*genes were mapped to grapevine chromosomes based on information available at the grape genome website (http://genomes.cribi.unipd.it/). The exon-intron organization of *VviSOS3* genes was determined by comparing predicted coding sequences with their corresponding genomic sequences using GSDS software (http://gsds.cbi.pku.edu.cn). The domain architecture of *VviSOS3* amino acid (AA) sequences were predicted using PlantsP (http://plantsp.genomics.purdue.edu/cgi-bin/fscan/feature_scan_rest.cgi), and ScanProsite software (http://prosite.expasy.org/scanprosite/).

### Preparation and treatment of plant material

*In vitro* grapevine plants (*V*. *vinifera*, ‘PN40024’, the sequenced genotype, inbred line of Pinot noir) were provided by Dr. Anne-Froncoise Adam-Blondom, INRA, France. This cultivar was always used as experimental materials for isolation and expression analysis of gene family in grapevine (*Vitis vinifera*) in response to abiotic stress [37–39]. Plants were maintained in 3/4 MS medium supplied with0.3 mg/L indole 3-butyric acid (IBA, Sigma USA), under a 16/8h photoperiod (100μmol m^-2^s^-1^) at 25°C in a tissue culture room.

NaCl treatment: Forty-day-old tissue cultured plants were placed into a Hoagland nutrient solution. Plants were allowed to adjust for 36h by which time they exhibited normal growth. The salt stress treatment was applied by adjusting the Hoagland nutrient solution to contain 250mM NaCl. Control plants were grown in Hoagland's nutrient solution only.

PEG stress treatment: The osmotic potential of the Hoagland nutrient solution was adjusted with PEG6000 (0.188g/ml) so that it was equivalent to the osmotic potential of the 250 mM NaCl treatment. Control plants were grown in Hoagland's nutrient solution without the addition of PEG.

Plants were kept in a growth chamber with a 16/8h photoperiod cycle, 25°Crelative humidity of 60–70%, and a light intensity of 100 μmol/m2/s. Leaves (2-8th leaf from the stem tip), stems, and roots were collected from control plants and the two treatments at 1, 4, 8, 12, 24, and 36 h, after the stress treatment were applied and used in all the subsequent analyses. Three biological replicates were collected for each treatment and the control.

### Reverse transcription quantitative PCR (RT-qPCR)

Total RNA was extracted from the grapevine samples using a Quick RNA isolation Kit (Cat.No.ZH120; Huayueyang Biotechnology, Beijing, China), following the manufacturer’s protocols. A traditional digestion step utilizing rDNase (TaKaRa Biotechnology, Dalian, China) was performed to ensure there was no DNA contamination in the samples. The quality and quantity of RNA extracted from each sample were determined using agarose gel electrophoresis and a Nanodrop ND-1000 Spectrophotometer (Thermo Fisher Scientific Inc.; USA), respectively. For cDNA synthesis, 500 ng high-quality RNA was reverse transcribed with oligdT and random primers using Super Script III Reverse Transcriptase (TaKaRa, Dalian, China) according to the manufacturer’s instructions. cDNA was diluted to approximately 100 ng/μl with ddH2O for the RT-qPCR analyses.

*VviSOS3*gene-specific primers were designed for the 3’-untranslated region and 3’terminal sequences of the predicted coding region using Beacon Designer 7.0 software (Premier Biosoft International, Palo Alto, CA, USA). All of the primers were tested and validated by PCR amplification to ensure their specificity (Table 1).

**Table 1:** Sequence of primers and PCR amplified products.

Gene name	Sequence	Amplified fragment length
*VviSOS3*.*1*	F:5’-AGGACAGACAGGCTTCAT-3’ R:5’-TAGTATGGATGGATTCCGAGAA-3’	199
*VviSOS3*.*2*	F:5’-ATCAATCGTGGATAAGACAATGGCAGAG-3’ R:5’-ACCTTCGGTGACGGAGATGGATG-3’	235
*VviSOS3*.*3*	F:5’-AGAGGTGGATGAGATTGCTA-3’ R:5’-CCGACAAGTGAGAACTACTG-3’	212
*VviSOS3*.*4*	F:5’-CTGATGCTGATGCTGACAA-3’ R:5’-TGAGTAGAATTGACAACACAGAG-3’	274
*VviSOS3*.*5*	F:5’-ATTCTGAGGTTGAGGACTGA-3’ R:5’-CTTGAGTGATGCTGCTATTCT-3’	184
*VviSOS3*.*6*	F:5’-GACAATGCCACTCTATCTGAAT-3’ R:5’-TGCTACTTCTTCCTTGGTAATG-3’	243
*VviSOS3*.*7*	F:5’-AGAATCCATCCCTGATAAAGAAC-3’ R:5’-TAGTCCTTGCTGCTGTCA-3’	226
*VviSOS3*.*8*	F:5’-TACAAGAAGCCCTAGCAGATGAATC-3’ R:5’-AAGCCTTTCTCCAGTCGGTTCC-3’	150
*VviSOS3*.*9*	F:5’-TTGCTGAATCTGGTATGAATCTT-3’ R:5’-TGGTGGTGATGTCCTTGA-3’	183

RT-qPCR was carried out on ABI PRISM 7300 Real-time PCR System (Applied Biosystems, Foster City, CA USA) using SYBR Premix Ex TaqTM (TaKaRa Code: DRR420A, TaKaRa, Dalian, China). The reaction mixture (total volume of 20 μl) contained 10 μl SYBR Premix Ex TaqTM(2×), 0.4 μl of each primer(10 μM), 0.4 μl ROX Reference Dye(50×), and 1 μl of template(about 100ng/μl).The total volume was adjusted to 20 μl by adding ddH2O. The PCR program was composed of the following steps: 95°C /30 s for pre-denaturation (step1), 95°C/5 s for denaturation (step2), 60°C/34s for primer annealing/extension and obtaining the fluorescent signal (step 3), for 40 cycles. Each biological replicate was analyzed in triplicate to ensure the accuracy of the results. A melting curve analysis was also conducted in order to verify the specificity of each primer pair utilizing the following program: 95°C/15s, 60°C/1min, 95°C/15s. The house-keeping gene (*actin101-like*, *VIT_12s0178g00200*) was used as a reference gene for qPCR [40].

Relative gene expression was quantified using the 2^-ΔΔC^ method [△△Ct = (Ct_target gene_—Ct_actin gene_) treatment- (Ct_target gene_−Ct_actin gene_) control]. All data were normalized based on the level of relative expression level in order to determine fold differences. The expression level of the various SOS genes at 0-h in the salt and PEG treated stem tissue was set as “1”. Expression levels “Above 1” or “below 1” in all other samples were considered as up- or down-regulated, respectively.

### Physiological parameters

Proline content was estimated by placing 0.2–0.5 g of fresh sample into a centrifuge tube along with 10 ml 3% sulfosalicylic acid. The tube was then placed in boiling water for 10 min. The samples were then centrifuged for 10 min (4000 r/min at 4 ^o^C). After centrifugation, 2 ml of supernatant, 2 ml acetic acid, and 2 ml 2.5% indene were added to a glass test tube (in triplicate) and incubated in boiling water bath to allow the chromogenic reaction to develop for 30 min. The tubes were then cooled to room temperature and 4 ml toluene were added, and the tubes were shaken. The red material present in the toluene layer was extracted and absorbance at 520 nm was measured [41,21]. The soluble protein content of each sample was also measured as described by Irigoyen [42].

Leaf, root and stem samples were harvested at 0, 1, 4, 8, 12, 24, and 36 h from the NaCl (250 mM) treatment and dried thoroughly at 80°C for two days. The tissues were then digested in concentrated 2/1 (v/v) HNO3/HClO4. The Na^+^, K^+^, Ca^2+^ ion concentration was determined with ICP-MS (Iris Intrepid II; Thermo Electron Corporation, Franklin, MA, USA) as described by Krachler [43].

### Statistical analysis

The software SPSS version 13.0 (SPSS, Chicago, IL, USA) and EXCEL (Microsoft Corporation, Redmond, WA, USA) were used for the statistical analyses. All data were obtained from three independent experiments with three biological replicates, if applicable. P < 0.05 and P < 0.01 were taken as statistically significant or highly significant, respectively.

## Results

### Identification and annotation of SOS3 genes in the grapevine genome

**Fig 1:**
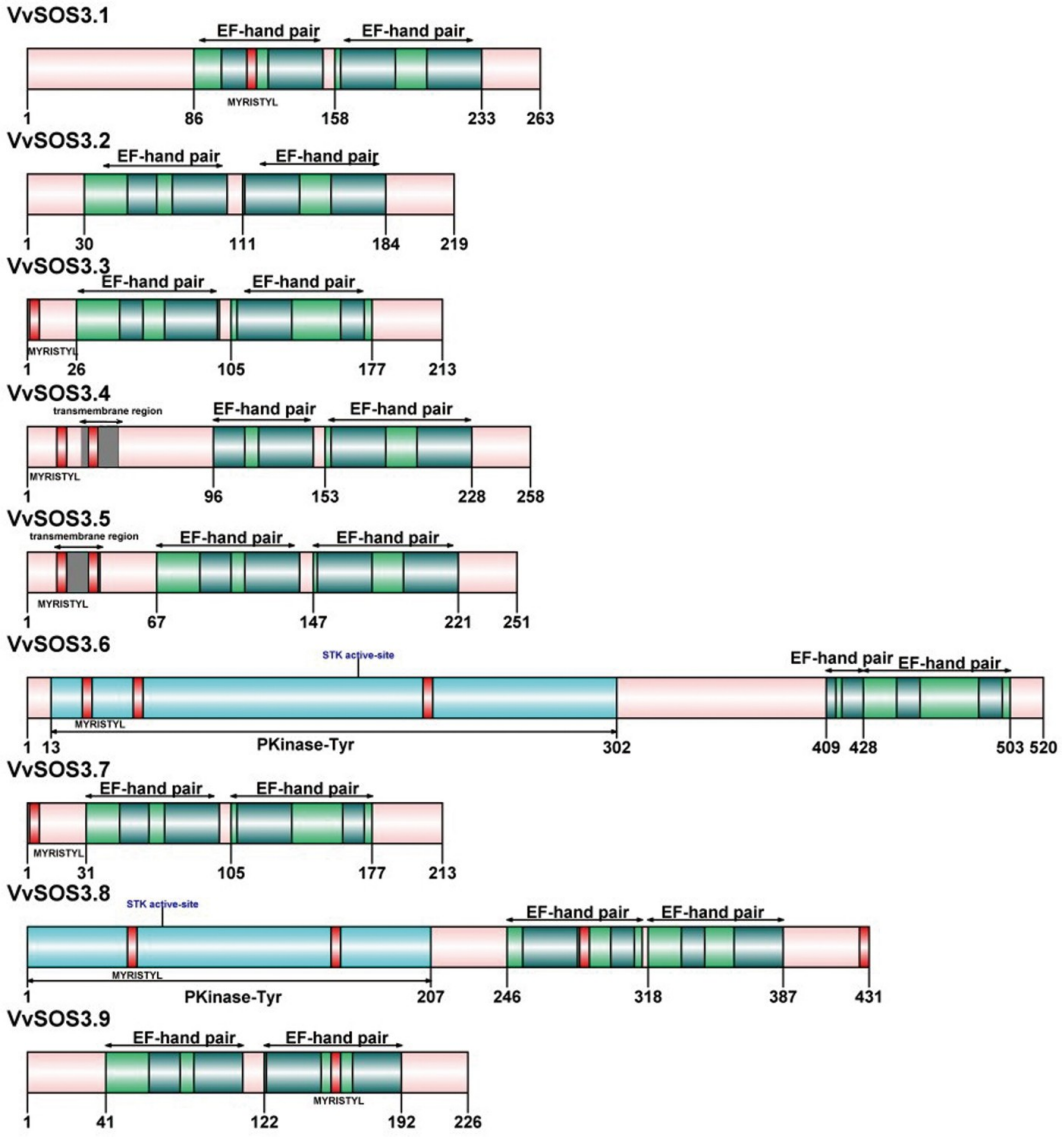
Diagrammatic illustration of the composition and domain distribution of VviSOS3 proteins. The domains are displayed in different colored boxes: gray, transmembrane region; blue, protein kinase-Tyr (Pkinase-Tyr); green, EF-hand; red, N-myristoylation.

A total of nine, full-length genes encoding putative SOS3 proteins were identified in the ‘PN40024’ grapevine genome (ver. 12X V1) available on the CRIBI Biotech website (http://genomes.cribi.unipd.it/). The nine *VviSOS3* genes were named *VviSOS3*.*1* to *VviSOS3*.*9* based on the order of the chromosomes (from low to high) on which they were located [44]. The parameters listed in Table 2 include the chromosome on which the gene is located, the predicted protein length, molecular weight, electric point, aliphatic index, grand average of hydropathicity (GRAVY), and the number of two functional motifs (EF hand and Myristoylation site) present in the protein and that are characteristic of SOS3 proteins. The nine *VviSOS3* genes are located on six of the nineteen grapevine chromosomes (Chr). Chr 2 possesses three *VviSOS3* genes and Chr4 has two. The other four genes are located on Chr13, 14, 16, and Chr19, respectively. *VviSOS3* proteins have a predicted protein length ranging from 213 (*VviSOS3*.*7)* to 520 (*VviSOS3*.*6*) amino acids, and a pI range of 4.68 (*VviSOS3*.*3*) to 5.62 (*VviSOS3*.*6*). The variability in the various parameters may be an indication that SOS3 genes with a similar pI may respond and function to the same environmental or cellular cues. *VviSOS3*.*6* and *VviSOS3*.*8* are the two longest proteins and have the highest pIs. GRAVY values indicate that *VviSOS3* genes encode hydrophilic proteins. In addition, all of the *VviSOS3* genes contained two EF-hand pair domains that function as Ca2+-binding sites (Table 2, Fig 1), and vary from 0–4 in the number of myristoylation sites.

**Table 2:** *SOS3* genes identified in the genome sequence of *V*. *vinifera*‘PN40024’.

Gene name	Gene ID	Chr	AA	MW (kDa)	pI	Ai	GRAVY	EF-hand pair	Myristoyla ton motif
*VviSOS3*.*1*	VIT_02s0025g 1630	2	263	30093.2	4.93	89.35	-0.143	2	1
*VviSOS3*.*2*	VIT_02s0025g 1640	2	219	25244.6	4.71	86.39	-0.276	2	0
*VviSOS3*.*3*	VIT_02s023g0 140	2	213	24472.8	4.68	90.61	-0.18	2	1
*VviSOS3*.*4*	VIT_04s0008g 950	4	258	29813	5.02	45.57	-0.166	2	2
*VviSOS3*.*5*	VIT_04s0008g 960	4	251	28988	4.88	39.77	-0.122	2	2
*VviSOS3*.*6*	VIT_13s0047g 260	13	520	57942.1	5.62	46.08	-0.229	2	3
*VviSOS3*.*7*	VIT_16s0098g 1870	16	213	24518.8	4.72	43.4	-0.28	2	1
*VviSOS3*.*8*	VIT_17s0000g 5520	17	431	49006.9	5.24	87.52	-0.337	2	4
*VviSOS3*.*9*	VIT_19s0015g 1070	19	226	26009.6	4.76	94.91	-0.215	2	1

Abbreviations: Chr, chromosome; AA, number of amino acids; number; MW, molecular weight; pI, isoelectric point; Ai,aliphatic index; GRAVY, grand average of hydropathicity; EF-hand pair, number of EF-hand pair domains; Myristoylation motif, number of predicted N-myristoylation sites.

### Phylogenetic analysis, classification, composition, and structure of *VviSOS3* genes

**Fig 2:**
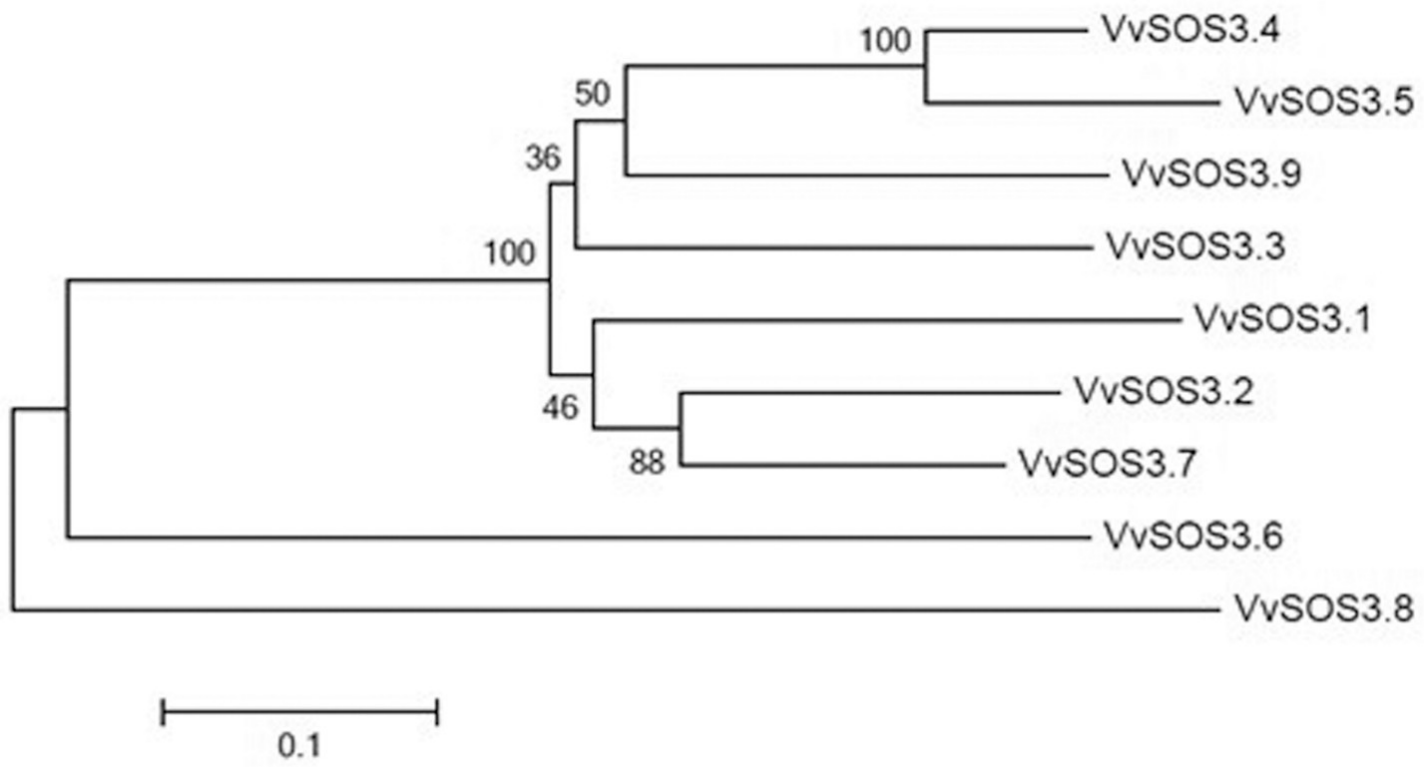
Phylogenetic tree of nine VviSOS3 genes. A neighbor-joining tree was constructed based on the alignments of the VviSOS3 genes with p-distance and 1,000 bootstrap repeats using MEGA 5.0 software.

A phylogenetic tree was constructed from the nine *VviSOS3* genes using the neighbor-joining method (Fig 2). The tree topology indicates that seven of the *VviSOS3* genes (*VviSOS3*.*1–5*, *7* and *9*) are more closely related to each other than the other two (*VviSOS3*.*6* and *8*) genes, both of which have a serine/threonine protein kinase (STK) active site (Fig 1). All of the *VviSOS3* genes have at least one conserved myristoylation site, with the exception of *VviSOS3*.*2* which has zero (Table 2, Fig 1). *VviSOS3*.*8* has the most myristoylation sites with a total of four. The myristoylation sites suggest that *VviSOS3* proteins are located on cell membranes, where they could act as part of a signal cascade in the SOS pathway [30,12]. Two other distinct motifs and transmembrane regions were identified in *VviSOS3*.*4*, *VviSOS3*.*5*, *VviSOS3*.*6* and *VviSOS3*.*8* uniquely possess a protein tyrosine kinase domain (PTK) and a serine/threonine protein kinase (STK) active site, which may explain their clustering together on a separate outer branch of the phylogenetic tree.

**Fig 3:**
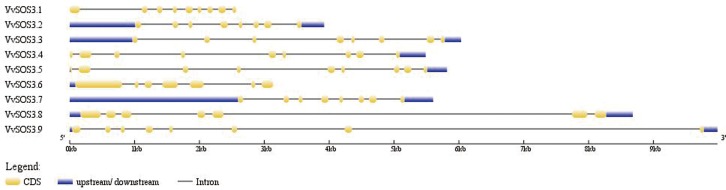
Diagrammatic illustration of exon/intron and UTR structure of thenine VviSOS3 genes. Gene IDs are indicated on the left. Regions of coding sequence (CDS) are represented by yellow boxes. Lines connecting CDS regions represent introns and untranslated regions (UTR) are represented by blue boxes. All of the designated regions are drawn to scale.

The exon-intron structure of the nine *VviSOS3* genes was analyzed in order to gain more insight into the evolution of the *VviSOS3* family in grapevine (Fig 3). Seven of the *VviSOS3* genes contain seven to eight introns, whereas *VviSOS3*.*6* and *VviSOS3*.*8* possess only six introns. *VviSOS3*.*8* and *VviSOS3*.*9* have a very large intron (more than 5kbp) located at their 3’ end, which greatly enhanced the differences in gene length among the *VviSOS3* genes. Two pairs of genes (*VviSOS3*.*4* and *VviSOS3*.*5*, *VviSOS3*.*2* and *VviSOS3*.*7*) possess a similar exon-intron structure. Moreover, *VviSOS3*.*4* and *VviSOS3*.*5* possess only one UTR region which is located at the 3’end of their sequences (Fig 3), while *VviSOS3*.*6* has only one UTR located at the 5’ end of its sequence. Six of the *VviSOS3* genes have two UTR regions located at each end of their sequences, while *VviSOS3*.*1* does not contain any UTR region (Fig 3).

### Expression pattern of the nine *VviSOS3* genes in response to salt and PEG treatments

**Fig 4:**
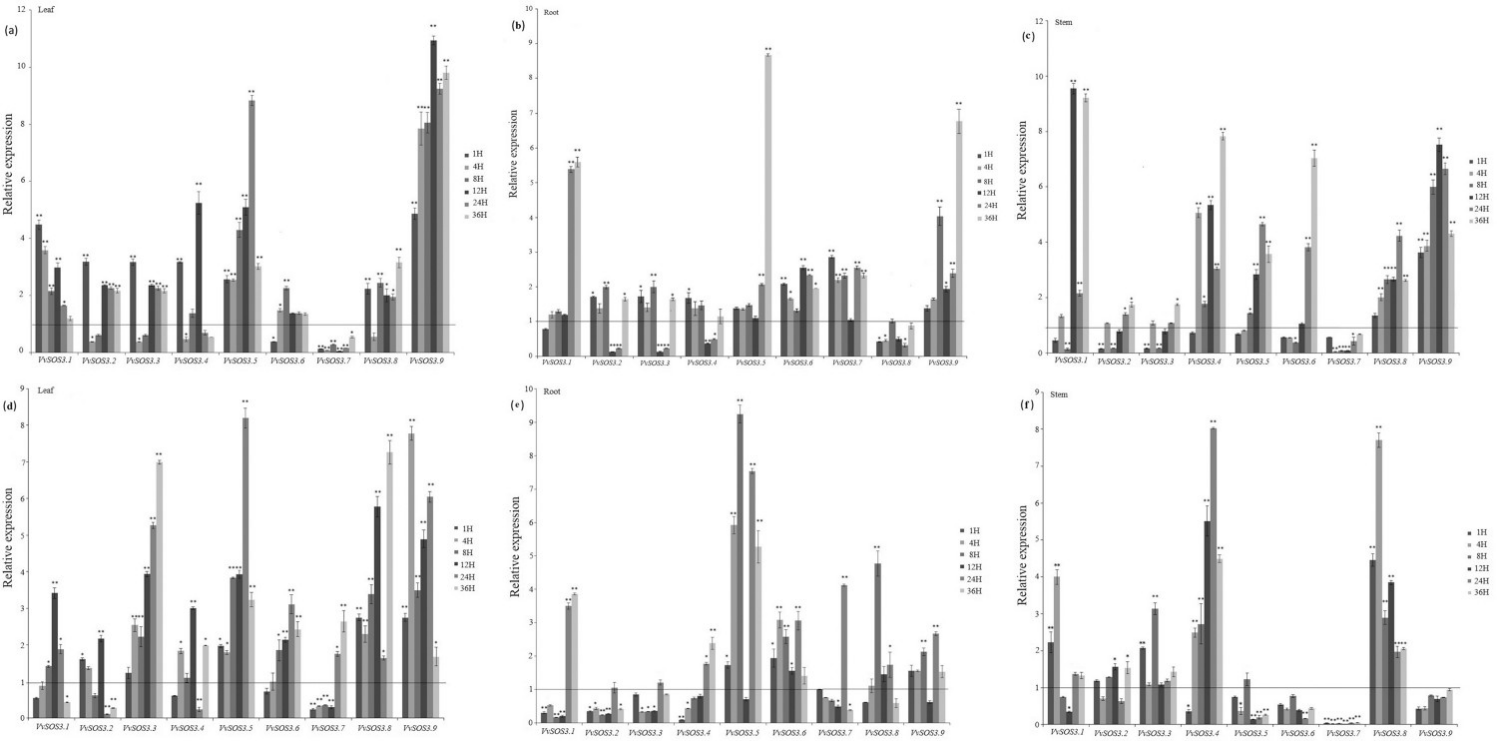
Expression profiles of VviSOS3 genes in grape (Vitis vinifera) leaves, roots, and stems in response to salt and polyethylene glycol (PEG) treatments as determined by RT-qPCR. (a—c) Relative expression of VviSOS3 genes in response to 18% polyethylene glycol (PEG) (PEG6000) stress treatment. (a) leaves; (b) roots; (c) stems. (d-f) Relative expression of VviSOS3 genes in response to NaCl (250mM) stress treatment, (d) leaves; (e) roots; (f) stems. RT-qPCR analysis of VviSOS3gene expression in leaves, roots and stems of four-week-old in vitro explants of V. vinifera ‘PN40024’. Tissues were collected at 1,4,8,12,24, and 36 h after the stress treatment was applied. Transcripts were normalized based on the expression of a grape actin gene. To visualize the relative expression levels data are presented as the mean fold changes between treated and control samples at each time point ± standard deviations (SDs). ** and * indicate significant differences in comparison with the control at P < 0.01 and P < 0.05, respectively.

The nine *VviSOS3* genes were differentially expressed in leaves, roots and stems in response to the PEG (Fig 4A–4C) and salt (Fig 4D–4F) treatments (Fig 4). Eight of the nine *VviSOS*3 genes were up-regulated to various degrees in leaves in response to the PEG treatment in at least one or more time points, with the highest expression reaching almost 11-fold in *VvSOS3*.*9* at the later time points (Fig 4A). In contrast, *VviSOS3*.*7* was significantly down-regulated at all the time points. All nine *VviSOS3* genes were up-regulated leaves in response to the NaCl treatment (Fig 4D), though the timing of their increased expression varied. These results indicate that all of the *VviSOS3* genes, except *VviSOS3*.*7* which was down-regulated, respond to PEG stress by being up-regulated and all were up-regulated in response to NaCl stress.

All of the *VviSOS3* genes, except *VviSOS3*.*8*, were up-regulated in roots in response to the PEG treatment (Fig 4B), and all of the *VviSOS3* genes, except *VviSOS3*.*2*, were up-regulated in response to the NaCl treatment (Fig 4E). All of the *VviSOS3* genes varied in the timing and level of their up-regulation. *VviSOS3*.*1* was up-regulated in response to both stresses at the later time points of sampling and exhibited a similar level of induction in response to both salt and PEG stress. Three genes (*VviSOS3*.*2*–*4*) exhibited a similar pattern in their expression levels in response to PEG stress, exhibiting a significant down-regulation at 12 h and 24 h, followed by up-regulation at 36 h. *VviSOS3*.*8* was generally down-regulated in response to PEG stress (Fig 4B) and up-regulated (Fig 4E) in response to salt stress.

All of the *VviSOS3* genes, except *VviSOS3*.*7*, were up-regulated in grapevine stems in response to PEG stress at different time points and to varying levels (Fig 4C), although six of them (*VviSOS3*.*1–6*) were down-regulated to varying degrees during the early sampling time points. *VviSOS3*.*6* and *VviSOS3*.*9* were consistently down-regulated in stem samples in response to the NaCl treatment (Fig 4F), while the expression of *VviSOS3*.*7* was down-regulated in response to both treatments (Fig 4C and 4F). While all of the *VviSOS3* genes, except *VviSOS3*.*7*, exhibited same level of up-regulation in response to the PEG treatment (Fig 4C); only six *VviSOS3* genes (*VviSOS3*.*1–5* and *VviSOS3*.*8*) exhibited significant levels of up-regulation, though to large varying degrees, in response to salt stress (Fig 4F).

### Physiological responses of grapevine ‘PN40024’ to salt and PEG stress

**Fig 5:**
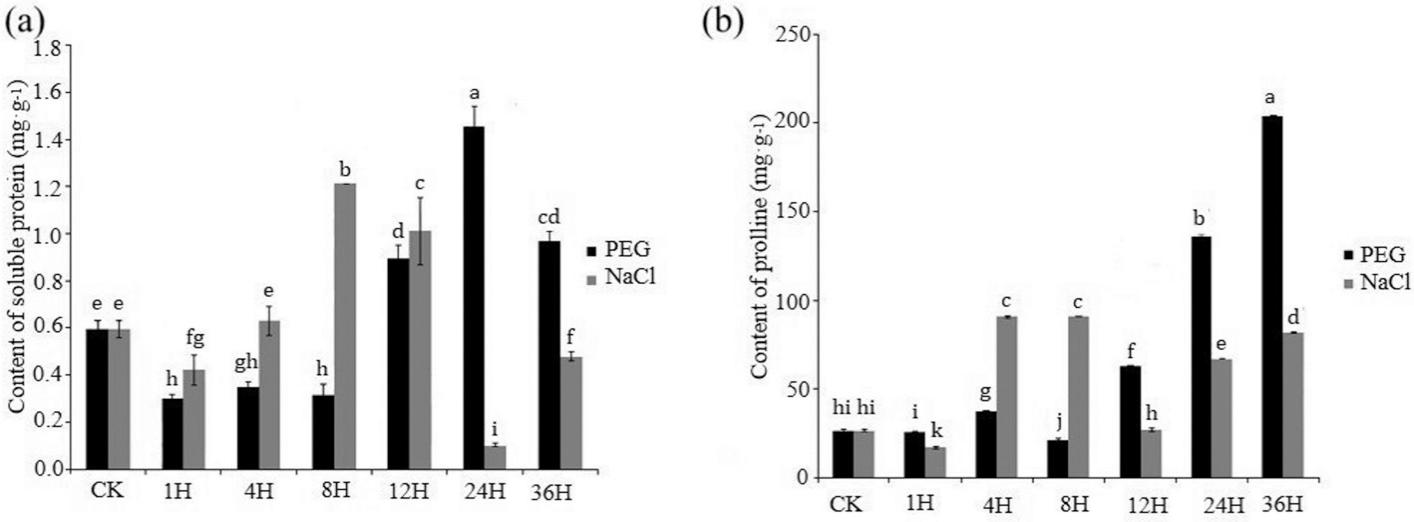
**Effect of PEG and salt (250mMNaCl) treatments on the soluble protein (a) and proline (b) content in leaves of grape (Vitis vinifera).** To visualize the relative expression levels data are presented as the mean fold changes between treated and control samples at each time point ± standard deviations (SDs).

Fig 5 shows the effect of NaCl and PEG treatment on soluble protein content and proline content in grape leaves. The soluble protein content in grape leaves had significant decreased at the first time (1H). Then the content of soluble protein in grape leaves under the NaCl treatment was higher than it under the PEG treatment, before 12H. However, the opposite occurred at 24H and 36H. In general, the soluble protein levels observed increases occurred more quickly in response to the salinity treatment (8h) than to the PEG treatment (12h) (Fig 5A). In response to PEG stress, the trends of proline content in leaves was unsteady at the early sampling times (0–8 h) and then achieved significant and sustained growth after 8H (Fig 5B). Meanwhile, proline levels increased significantly from 1H to 4H in response to salt stress, then declined significantly at 12H, which was subsequently followed by a substantial increase after 12H (Fig 5B).

**Fig 6:**
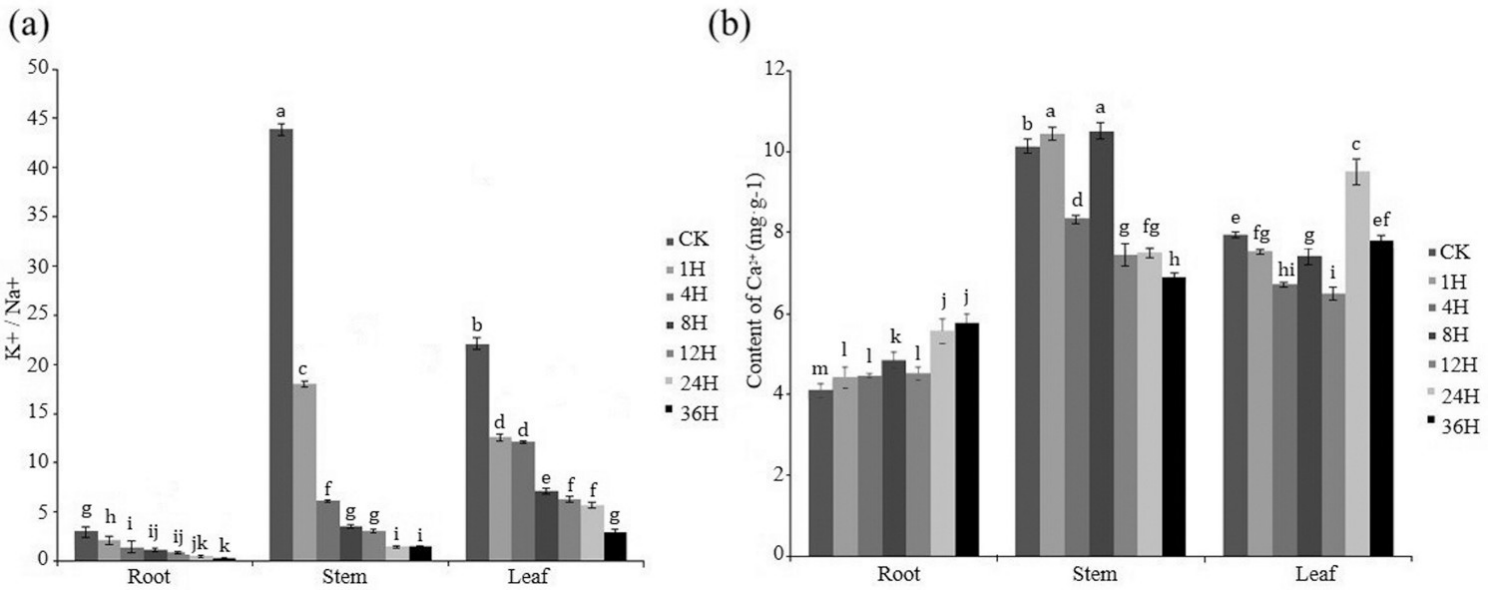
Effect of salt stress (250 mM NaCl) on the K+/Na+ ratio and calcium ion content in leaves, stem, and roots of grape (Vitis vinifera). (a) K+/Na+ ratio in leaves, stem and roots. (b) Ca2+content in leaves, stems, and roots. To visualize the relative expression levels data are presented as the mean fold changes between treated and control samples at each time point ± standard deviations (SDs).

The K^+^/Na^+^ ratio exhibited a continual decrease throughout the sampled time points in response to the salt treatment in all of the plant tissues (Fig 6A). Among the three tissues, stems maintained the highest K^+^/Na^+^ ratio, while roots exhibited the lowest ratio. In comparison to roots and leaves, the K^+^/Na^+^ ratio decreased much faster in stems (Fig 6A). Calcium content declined under salinity treatment in leaves at early stages, but increased dramatically at 24H (increased by 25%) (Fig 6B). Under the salt stress Ca^2+^ content increased significantly at 1H and 8 in stems, but decreased significantly at 4H and in later stages (after 8H) until the end of the treatment (almost 30% decreased at 36h). In root, Ca^2+^ was lower than those in leaves and stems. But it showed a steady increases trends during the treatment (Fig 6B).

## Discussion

### Identification and bioinformatic analysis of *VviSOS3* genes

The nine *VviSOS3* genes in *V*. *vinifera* cluster into two groups in the phylogenetic tree analysis (Fig 2). One cluster contains *VviSOS3*.*1*, *VviSOS3*.*2*, *VviSOS3*.*3*, *VviSOS3*.*4*, *VviSOS3*.*5*, *VviSOS3*.*7*, and *VviSOS3*.*9* and the other cluster contains *VviSOS3*.*6* and *VviSOS3*.*8* (Fig 2). The sequence differences between the *VviSOS3* genes in the two clusters suggest that a functional divergence may exist in *VviSOS3* genes. Our analyses also revealed that the more closely-related genes, such as *VviSOS3*.*3*, *VviSOS3*.*4*, *VviSOS3*.*5* and *VviSOS3*.*9*, tended to have a similar compositional structure (exon/intron, myristoylation sites, UTR location, etc.). The PTK motif has been reported to be involved in Ca^2+^-induced regulation of ion channels by transferring γ-phosphate groups from ATP to protein substrates [44]. Therefore, it could be inferred that the PTK motif present in *VviSOS3*.*6* and *VviSOS3*.*8* functions in perception and signal transduction.

All nine *VviSOS3* genes possess two pairs of EF-hand domains (Fig 1). This is similar to *Arabidopsis SOS3* genes, indicating that *VviSOS3* genes have the potential to bind Ca^2+^ [29]. The EF-hand type calcium-binding portion of the *VviSOS3* protein is similar to animal neuronal calcium sensors and the yeast calcineurin B sub-unit [12]. Eight of the nine *VviSOS3* proteins also have myristoylation sites (Fig 1). The EF-hand pair and N-myristoylation domains are considered the regions of SOS3 proteins that play a functional role in plant response to stress and N-myristoylation sites in *VviSOS3* proteins are essential for their interaction with and activation of their molecular partner, SOS2 [30]. The present study found that only *VviSOS3*.*2* lacked an N-myristoylation site, suggesting that some functionality may have been lost in this *VviSOS3* gene [30]. This particular deletion may be the basis of the down-regulated expression of *VviSOS3*.*2* in roots in response to salt stress (Fig 4E). Additionally, *VviSOS3*.*4* and *VviSOS3*.*5* both display a transmembrane region, which may be involved in membrane location and trans-membrane transport.

### *VviSOS3* gene expression

The results of the present study revealed both marked differences and similarities in the response and expression patterns of *VviSOS3* genes in leaves, stems, and roots in response to PEG and salt stress. It should be noted that the PEG treatment only induces an osmotic stress, whereas the salt treatment would induce both an ionic and an osmotic stress [45]. The well-defined, salt overly sensitive (SOS) signaling pathway is required for ion homeostasis [16]. While the three grapevine organs (stem, leaf, and root) exhibited varying patterns of both up-and down-regulation of *VviSOS3* genes at different time points, significantly more *VviSOS3* genes were up-regulated than down-regulated in response to the salt and PEG treatments. These observations indicate that the *VviSOS3* gene family may play a significant role in the response to both stresses. Transgenic *Arabidopsis* plants over-expressing SOS pathway genes have been reported to exhibit lower Na^+^ and higher K^+^ accumulation than wild-type plants in response to salt stress, resulting in higher salinity stress tolerance [46]. Similar results were observed in rice (*Oryza sativa*) where salt tolerance was associated with greater expression of *SOS1*, *SOS2* and *SOS3* and the higher expression of these genes was correlated with the ability to exclude Na^+^ from the shoot and maintain a low cellular Na^+^/K^+^ratio [21]. Most of the *VviSOS3* genes were regulated in a similar manner in the three organs in response to both PEG and salt stress. These results suggest that *VviSOS3* genes are potentially involved in establishing and maintaining ionic and osmotic homeostasis. Our results also indicated that there were also similarities in the expression pattern of *VviSOS3* genes in the three different grapevine organs. In general, however, the present study also indicated that *VviSOS3* genes were regulated in a tissue-specific manner, suggesting that these genes are part of similar but distinctly different responses designed to cope with salt and PEG stress.

In response to the PEG and salt treatments, *VviSOS3*.*7* exhibited a different expression pattern leaves and stems related to the other eight *VviSOS3* genes (Fig 4A and 4C). *VviSOS3*.*7* was down-regulated in leaves and stems and was up-regulated in root in response to PEG, however, it was up-regulated in leaves and stems, and was down-regulated in root in response to salt stress. This suggests that the regulation mechanism of *VviSOS3*.7 was affected by ion stress. The *VviSOS3*.7 is a positive regulator in the response of aboveground parts to PEG stress and is a negative regulator in response to salt treatment.

*VviSOS3*.*2* and *VviSOS3*.*3*, exhibited similar expression patterns in response to the PEG treatment. The two genes were significantly down-regulated at 4h and 8h in leaves and were significantly down-regulated at 12h and 24h, and were significantly down-regulated at 1h and 8h, which may suggest that the functions of the two genes may be very similar in regulating and adjusting the response to osmotic stress. *VviSOS3*.*5*, *VviSOS3*.*6*, and *VviSOS3*.*9* exhibited elevated expression levels in response to PEG, but reduced levels of expression in stems in response to NaCl (Fig 4C and 4F). *VviSOS3*.*8* was up-regulated in root in response to salt stress, but was down-regulated in root in response to PEG (Fig 4B and 4E), which supports the potential involvement of *VviSOS3*.*8* in maintaining ionic homeostasis in response to salt stress. In contrast, it could also be inferred that *VviSOS3*.*5*, *VviSOS3*.*6*, and *VviSOS3*.*9* play a specific role in regulating osmotic homeostasis in response to PEG stress.

All the nine genes, only *VvSOS3*.*7* and *VvSOS3*.*8* expressed the opposite expression pattern in response to PEG stress and salt stress. *VviSOS3*.*7* was down-regulated in aboveground parts and was up-regulated in underground parts in response to PEG, however, it just was up-regulated in aboveground parts, and was down-regulated in underground parts in response to salt stress. Compared with *VviSOS3*.*7*, *VviSOS3*.*8* performance on the contrary (Fig 4B and 4E). However, the PEG treatment we used was equivalent to the osmotic potential of the NaCl treatment we used, the ion stress of NaCl is the only difference. Therefore we inferred that the differences expression of *VviSOS3*.*7* and *VviSOS3*.*8* to PEG and NaCl stress were probably caused by the ion stress.

### Physiological parameters

The ability to maintain osmotic homeostasis in plants is critical when they are subjected to abiotic stresses that result in large changes in the ionic balance [47]. Adjustments in intracellular soluble proteins and proline can contribute to maintain cell water potential and turgor pressure, thus enhancing the ability of plants to tolerate drought and salinity stress [48]. Proline is one of the main osmotically-active compounds used by plants to regulate cytoplasmic and vacuolar water relations in response to salt stress [49]. Our study revealed that proline increased gradually in grape in response to both salt and PEG stress (Fig 5B), suggesting that proline is involved in the regulation of osmotic homeostasis.

Soluble proteins are also important osmotic regulators when plants are subjected to adverse environmental conditions [50]. Results of the present study revealed that the soluble protein content in grape leaves also increased substantially in response to both PEG and salt stress, though significantly more in response to PEG than to salt stress (Fig 5A). These data indicate that soluble proteins play an important role in osmotic adjustment. This contribution, however, appears to have limitations as the soluble protein content decreased in grape leaves at the later sampling times, which may have been due to ionic-based injury to membranes caused by the salt stress.

The K^+^/Na^+^ ratio (Fig 6A), an established indicator for response to salt stress, decreased over time in stems, roots, and leaves of grape in response to the salt treatment; with the greatest decrease occurring in stem tissues. A hyper saline environment induces a perturbation in the ionic steady state of not only Na^+^ and Cl^-^, but also K^+^ and Ca^2+^ [45]. High NaCl causes the cytosolic accumulation of Ca^2+^ which is transported from the apoplast and intracellular compartments [51]. In our study, Ca^2+^ content increased gradually over time in roots in response to the salt treatment (Fig 6B), with a corresponding change in the expression level of *VviSOS3*.*1* and *VviSOS3*.*4*. Based on these data, we infer the active of *VviSOS3*.*1* and *VviSOS3*.*4* as the calcium sensors more active in the response of roots to salt stress.

Our results also indicated a significant reduction in Ca^2+^ content in stems (Fig 6B), where four where four *VviSOS3* genes were up-regulated (Fig 4F). These data suggest that four of the *VviSOS3* genes (*VviSOS3*.5, *VviSOS3*.6, *VviSOS3*.8 and *VviSOS3*.9) in different organs different calcium sensors express different effects in response to salt stress. Ca^2+^ has been widely implicated as an intracellular messenger in physiologically and environmentally-induced signaling pathways in plants [52]. An external supply of Ca^2+^ can improve salt tolerance by adjusting K^+^/Na^+^ selectivity [53,12]. Salt may trigger changes in the cellular Ca^2+^ signature (for example, an oscillation in the concentration of free cytosolic Ca^2+^), which is then perceived by various intracellular Ca^2+^ sensors/binding proteins that are responsible for regulating a variety of signaling cascades. For example, *SOS3* possesses an EF-hand type calcium-binding domain and physically interacts with and activates *SOS2* [54]. The calcium content in leaf and stems of grape, however, declined in response to the salt treatment, which may be due to the inhibition of ion uptake directly caused by the salt stress. Additional, studies will be required to reveal the specific function of each of the *VviSOS3* genes in the response of grape to osmotic stress in grape plants. The present study, however, provides a good foundation for designing future experiments.

Our study systemically determined the expression of grapevine *SOS3* genes in response to equal molar NaCl and polyethylene glycol (PEG) stress in leaves, stems, and roots of grape. Nine *VviSOS3* genes were identified in the 12XV1 version of the grapevine genome. The identified *VviSOS3* genes were differentially expressed in leaves, roots, and stems in response to NaCl and PEG treatments. And we identified the *VviSOS3*.*7* and *VviSOS3*.*8* as candidate genes for further functional characterizations regarding their role in the response of grapevine to salt stress and that will provide a foundation for understanding the role of the SOS pathway in grape.

## Supporting information

S1 Table Relative expression level of VviSOS3 genes in roots under NaCl (250mM) stress treatment. All data were obtained from three independent experiments with three biological replicates.
(XLSX)

S2 Table Relative expression level of VviSOS3 gene in stems under 18% polyethylene glycol (PEG) (PEG6000) stress. All data were obtained from three independent experiments with three biological replicates.
(XLSX)

S3 Table Determination of K+/Na+ ratio and calcium ion content in grape leaves, stem, and roots of grape (Vitis vinifera) treated with PEG and salt (250mMNaCl). a, Determination of K+/Na+ ratio in grape leaves at 0h, 4h, 8h, 12h, 24h and 36h under PEG and salt treatment (250mMNaCl). b, Determination of calcium ion content in grape leaves at 0h, 4h, 8h, 12h, 24h and 36h under PEG and salt treatment (250mMNaCl). All data were obtained from three independent experiments with three biological replicates.
(XLSX)

S4 Table Relative expression level of VviSOS3 gene in roots under 18% polyethylene glycol (PEG) (PEG6000) stress. All data were obtained from three independent experiments with three biological replicates.
(XLSX)

S5 Table Relative expression level of VviSOS3 genes in stems under NaCl (250mM) stress treatment. All data were obtained from three independent experiments with three biological replicates.
(XLSX)

S6 Table Relative expression level of VviSOS3 genes in leaves under NaCl (250mM) stress treatment. All data were obtained from three independent experiments with three biological replicates.
(XLSX)

S7 Table Determination of soluble protein and proline in grape leaves treated with PEG and salt (250mMNaCl). a, Determination of soluble protein content in grape leaves at 0, 4, 8, 12, 24 and 36h under PEG and salt treatment (250mMNaCl). b,Determination of proline content in grape leaves at 0, 4, 8, 12, 24 and 36h under PEG and salt treatment (250mMNaCl). All data were obtained from three independent experiments with three biological replicates.
(XLSX)

## References

[1] Wang M, Vannozzi A, Wang G, Liang YH, Tornielli GB, Zenoni S, et al (2014) Genome and transcriptome analysis of the grapevine (Vitis vinifera L.) WRKY gene family. Horticulture Research 1:14016 10.1038/hortres.2014.16 26504535 PMC4596322

[2] Chapman DM, Matthews MA, Guinard JX (2004) Sensory attributes of Cabernet Sauvignon wines made from vines with different crop yields. Am J Enol Vitic 55: 325–334.

[3] Roby G, Harbertson JF, Adams DA, Matthews MA (2004) Berry size and vine water deficits as factors in winegrape composition: Anthocyanins and tannins. Australian Journal of Grape and Wine Research 10: 100–107.

[4] Guo Y, Yang YQ (2018) Unraveling salt stress signaling in plants Journal of Integrative. Plant Biology 60(9): 796–804. 10.1111/jipb.12689 29905393

[5] Shani Uri, Alon Ben-Gal (2005) Long-term Response of Grapevines to Salinity: Osmotic Effects and Ion Toxicity. Am J Enol Vitic 56: 148–154.

[6] Zhu JK (2001) Plant salt tolerance. Trends in Plant Science 6: 66–71. 11173290 10.1016/s1360-1385(00)01838-0

[7] Yıldırım K, Yağci A, Sucu S, Tunç S (2018) Responses of grapevine rootstocks to drought through altered root system architecture and root transcriptomic regulations. Plant Physiology and Biochemistry 127: 256–258. 10.1016/j.plaphy.2018.03.034 29627732

[8] Sucu S, Yağci A, Yıldırım K (2018) Changes in morphological, physiological traits and enzyme activity of grafted and ungrafted grapevine rootstocks under drought stress. Erwerbs-Obstbau 60(2): 127–136.

[9] Mohammadkhani N, Heidari R, Abbaspour N, Rahmani F (2018) Effects of Salinity on Plant Hormones Genes in Grape Iranian. Journal of Science and Technology 42: 401–410.

[10] Blumwald E, Aharon GS, Apse MP (2000) Sodium transport in plant cells Biochim Biophys Acta 1465: 140–151. 10748251 10.1016/s0005-2736(00)00135-8

[11] Gaxiola RA, Rao R, Sherman A, Grisafi P, Alper SL, Fink GR (1999) The Arabidopsis thaliana proton transporters, AtNhx1 and Avp1, can function in cation detoxification in yeast. Proc Natl Acad Sci USA 96: 1480–1485. 9990049 10.1073/pnas.96.4.1480 PMC15488

[12] Liu J, Zhu JK (1998) A calcium sensor homolog required for plant salt tolerance. Science 280: 1943–1945. 9632394 10.1126/science.280.5371.1943

[13] Tsugane K, Kobayashi K, Niwa Y, Ohba Y, Wada K, Kobayashi H (1999) A recessive Arabidopsis mutant that grows photoautotrophically under salt stress shows enhanced active oxygen detoxification. Plant Cell 11: 1195–1206. 10402422 10.1105/tpc.11.7.1195 PMC144266

[14] Ishitani M, Liu J, Halfter U, Kim CS, Shi W, Zhu JK (2000) SOS3 function in plant salt tolerance requires N-myristoylation and calcium binding. Plant Cell 12: 1667–1678. 11006339 10.1105/tpc.12.9.1667 PMC149077

[15] Xi Y, Liu J, Dong C, Cheng Z-M (2017) The CBL and CIPK Gene Family in Grapevine (Vitis vinifera): Genome-Wide Analysis and Expression Profiles in Response to Various Abiotic Stresses. Frontiers in Plant Science 8: 978 10.3389/fpls.2017.00978 28649259 PMC5465270

[16] Qiu QS, Guo Y, Quintero FJ, Pardo JM, Schumaker KS, Zhu JK (2004) Regulation of vacuolar Na+/H+ exchange in Arabidopsis thaliana by the salt-overly-sensitive (SOS) pathway. Journal of Biological Chemistry 279 (1): 207–215. 10.1074/jbc.M307982200 14570921

[17] Fujii H, Zhu JK (2009) An autophosphorylation site of the protein kinase SOS2 is important for salt tolerance in Arabidopsis. Molecular Plant 2: 183–190. 10.1093/mp/ssn087 19529820 PMC2639731

[18] Ji H, Pardo JM, Batelli G, Van Oosten MJ, Bressan RA, Li X (2013) The Salt Overly Sensitive (SOS) pathway: established and emerging roles. Molecular Plant 6: 275–286. 10.1093/mp/sst017 23355543

[19] Guo Y, Qiu QS, Quintero FJ, Pardo JM, Ohta M, Zhang C, et al (2004) Transgenic evaluation of activated mutant alleles of SOS2 reveals a critical requirement for its kinase activity and C-terminal regulatory domain for salt tolerance in Arabidopsis thaliana. Plant Cell 16: 435–449. 10.1105/tpc.019174 14742879 PMC341915

[20] Ma DM, Wr WX, Li HW, Jin FX, Guo LN, Wang J, et al(2014) Co-expression of the Arabidopsis SOS genes enhances salt tolerance in transgenic tall fescue (Festuca arundinacea Schreb.). Protoplasma 251: 219–231. 10.1007/s00709-013-0540-9 24022678 PMC3893463

[21] Martinez-Atienza J, Jiang XY, Garciadeblas B, Mendoza I, Zhu JK, Pardo JM, et al (2007) Conservation of the salt overly sensitive pathway in rice. Plant Physiology 143: 1001–1012. 10.1104/pp.106.092635 17142477 PMC1803719

[22] Tang RJ, Liu H, Bao Y, Lv QD, Yang L, Zhang HX (2010) The woody plant poplar has a functionally conserved salt overly sensitive pathway in response to salinity stress. Plant Molecular Biology 74: 367–380. 10.1007/s11103-010-9680-x 20803312

[23] Zhu JK, Liu J, Xiong L (1998) Genetic analysis of salt tolerance in arabidopsis. Evidence for a critical role of potassium nutrition. Plant Cell 10: 1181–1191. 9668136 10.1105/tpc.10.7.1181 PMC144057

[24] Nutan KK, Kumar G, Singla-Pareek SL, Pareek A (2018) A Salt Overly Sensitive Pathway Member from Brassica juncea BjSOS3 Can Functionally Complement Delta Atsos3 in Arabidopsis. Current Genomics 19 (1): 60–69. 10.2174/1389202918666170228133621 29491733 PMC5817878

[25] Fahmideh L, Fooladvand Z (2018) Isolation and Semi Quantitative PCR of Na + /H + Antiporter (SOS1 and NHX) Genes under Salinity Stress in Kochia scoparia. Biological Procedures Online 20(1): 11. 29881329 10.1186/s12575-018-0076-7 PMC5984343

[26] Sathee L, Sairam RK, Chinnusamy V, Jha SK (2015) Differential transcript abundance of salt overly sensitive (SOS) pathway genes is a determinant of salinity stress tolerance of wheat. Acta Physiologiae Plantarum 37 (8): 169.

[27] Yousefirad S, Soltanloo H, Ramezanpour SS, Zaynalinezhad K, Shariati V (2018) Salt oversensitivity derived from mutation breeding improves salinity tolerance in barley via ion homeostasis. Biologia Plantarum 62(4): 775–785.

[28] Zhu JK (2000) Genetic Analysis of Plant Salt Tolerance Using Arabidopsis. Plant Physiology 124: 941–948. 11080272 10.1104/pp.124.3.941 PMC1539290

[29] Guo Y, Halfter U, Ishitani M, Zhu JK (2001) Molecular characterization of functional domains in the protein kinase SOS2 that is required for plant salt tolerance. Plant Cell 13: 1383–1400. 11402167 10.1105/tpc.13.6.1383 PMC135579

[30] Ishitani M, Liu J, Halfter U, Kim CS, Shi W, Zhu JK (2000) SOS3 function in plant salt tolerance requires N-myristoylation and calcium binding. Plant Cell 12: 1667–1678. 11006339 10.1105/tpc.12.9.1667 PMC149077

[31] Ye J, Zhang W, Guo Y (2013) Arabidopsis SOS3 plays an important role in salt tolerance by mediating calcium-dependent microfilament reorganization. Plant Cell Rep 32:139–148. 10.1007/s00299-012-1348-3 23052592

[32] Zhao YK, Wang T, Zhang WS, Li X (2011) SOS3 mediates lateral root development under low salt stress through regulation of auxin redistribution and maxima in Arabidopsis. New Phytologist 189: 1122–1134. 10.1111/j.1469-8137.2010.03545.x 21087263

[33] Shang G, Li Y, Hong Z, Liu CL, Shao-Zhen HE, Liu QC (2012) Overexpression of SOS Genes Enhanced Salt Tolerance in Sweetpotato. Journal of Integrative Agriculture 11: 378–386.

[34] Zhang HC, Yin WL, Xia XL (2008) Calcineurin B-Like family in Populus: comparative genome analysis and expression pattern under cold, drought and salt stress treatment. Plant Growth Regulation 56(2): 129–140.

[35] Aiyar A (1999) The Use of CLUSTAL W and CLUSTAL X for Multiple Sequence Alignment. Methods in Molecular Biology, 132: 221–241. 10.1385/1-59259-192-2:221 10547838

[36] Tamura K, Peterson D, Peterson N, Stecher G, Nei M, Kumar S (2011) MEGA5: molecular evolutionary genetics analysis using maximum likelihood, evolutionary distance, and maximum parsimony methods. Molecular Biology & Evolution 28: 2731–2739. 21546353 10.1093/molbev/msr121 PMC3203626

[37] Wang G, Lovato A, Polverari A, Wang M, Liang Y-H, Ma Y-C, et al (2014) Genome-wide identification and analysis of mitogen activated protein kinase kinase kinase gene family in grapevine (Vitis vinifera). Bmc Plant Biology 14: 219 10.1186/s12870-014-0219-1 25158790 PMC4243721

[38] Liu J, Chen N, Chen F, Cai B, Dal Santo S, Tornielli GB, et al (2014) Genome-wide analysis and expression profile of the bZIP transcription factor gene family in grapevine (Vitis vinifera). Bmc Genomics 15. 10.1186/1471-2164-15-281 PMC4023599 24725365

[39] Ma Y, Wang J, Zhong Y, Geng F, Cramer GR, Cheng Z-M (2015) Subfunctionalization of cation/proton antiporter 1 genes in grapevine in response to salt stress in different organs. Horticulture Research 2: 15031 10.1038/hortres.2015.31 26504576 PMC4591679

[40] Reid KE, Olsson N, Schlosser J, Peng F, Lund ST (2006) An optimized grapevine RNA isolation procedure and statistical determination of reference genes for real-time RT-PCR during berry development. Bmc Plant Biology 6(1): 27. 17105665 10.1186/1471-2229-6-27 PMC1654153

[41] Bates LS, Waldren RP, Teare ID (1973) Rapid determination of free proline for water-stress studies.Plant Soil 39: 205–207.

[42] Irigoyen JJ, Einerich DW, Sánchez-Díaz M (1992) Water stress induced changes in concentrations of proline and total soluble sugars in nodulated alfalfa (Medicago sativd) plants. Physiologia Plantarum 84: 55–60.

[43] Krachler M, Mohl C, Emons H, Shotyk W (2002) Influence of digestion procedures on the determination of rare earth elements in peat and plant samples by USN-ICP-MS. Journal of Analytical Atomic Spectrometry 17: 844–851.

[44] Vannozzi A, Dry IB, Fasoli M, Zenoni S, Lucchin M (2012) Genome-wide analysis of the grapevine stilbene synthase multigenic family: genomic organization and expression profiles upon biotic and abiotic stresses. Bmc Plant Biology 12: 1–22. 10.1186/1471-2229-12-1 22863370 PMC3433347

[45] Niu XM, Bressan RA, Hasegawa PM, Pardo JM (1995) Ion Homeostasis In Nacl Stress Environments. Plant Physiology 109 (3): 735–742. 12228628 10.1104/pp.109.3.735 PMC161372

[46] Yang Q, Chen ZZ, Zhou XF, Yin HB, Li X, Xin XF, et al (2009) Overexpression of SOS (Salt Overly Sensitive) Genes Increases Salt Tolerance in Transgenic Arabidopsis. Molecular Plant 2: 22–31. 10.1093/mp/ssn058 19529826 PMC2639737

[47] Rong SY, Guo SG, Zhang T (2011) Effects of Drought Stress on Osmoregulation Substances in Sweet Sorghum Seedlings. Journal of Henan Agricultural Sciences.

[48] Liu JX, Wang JC, Wang RJ, Jia HY (2012) Interactive Effects of Drought and Salinity Stresses on Growth and Osmotica of Naked Oat Seedlings. Journal of Soil & Water Conservation 26: 244–248.

[49] Stewart GR, Lee JA (1974) The role of proline accumulation in halophytes. Planta 120: 279–289. 10.1007/BF00390296 24442703

[50] Heyser JW, Nabors MW (1981) Growth, water content, and solute accumulation of two tobacco cell lines cultured on sodium chloride, dextran, and polyethylene glycol. Plant Physiology 68: 1454–1459. 16662125 10.1104/pp.68.6.1454 PMC426120

[51] Knight H, Trewavas AJ, Knight MR (1997) Calcium signalling in Arabidopsis thaliana responding to drought and salinity. Plant Journal for Cell & Molecular Biology 12: 1067–1078. 9418048 10.1046/j.1365-313x.1997.12051067.x

[52] Trewavas AJ, Rui M (1998) Ca2+ signalling in plant cells: the big network! Current Opinion in Plant 1(5): 428–433. 10.1016/s1369-5266(98)80268-9 10066614

[53] Cramer GR, Lynch J, Läuchli A, Epstein E (1987) Influx of Na+, K+, and Ca2+ into Roots of Salt-Stressed Cotton Seedlings: Effects of Supplemental Ca2+. Plant Physiology 83: 510–516. 16665280 10.1104/pp.83.3.510 PMC1056396

[54] Zielinski RE (1998) Calmodulin and calmodulin-binding proteins in plants. Annual Review of Plant Physiology & Plant Molecular Biology 49: 697–725. 10.1146/annurev.arplant.49.1.697 15012251

